# Supplementary figures and images for: The association between maternal infection and intellectual disability in children: A systematic review and meta-analysis
Source: PLoS One. 2023 Oct 5;18(10):e0292226. doi: 10.1371/journal.pone.0292226 (PMC10553326; doi:10.1371/journal.pone.0292226)

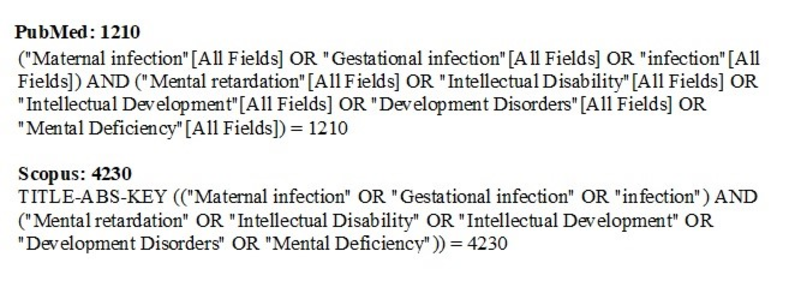

Supplement: S1 Fig — (TIF) [file pone.0292226.s002.tif]

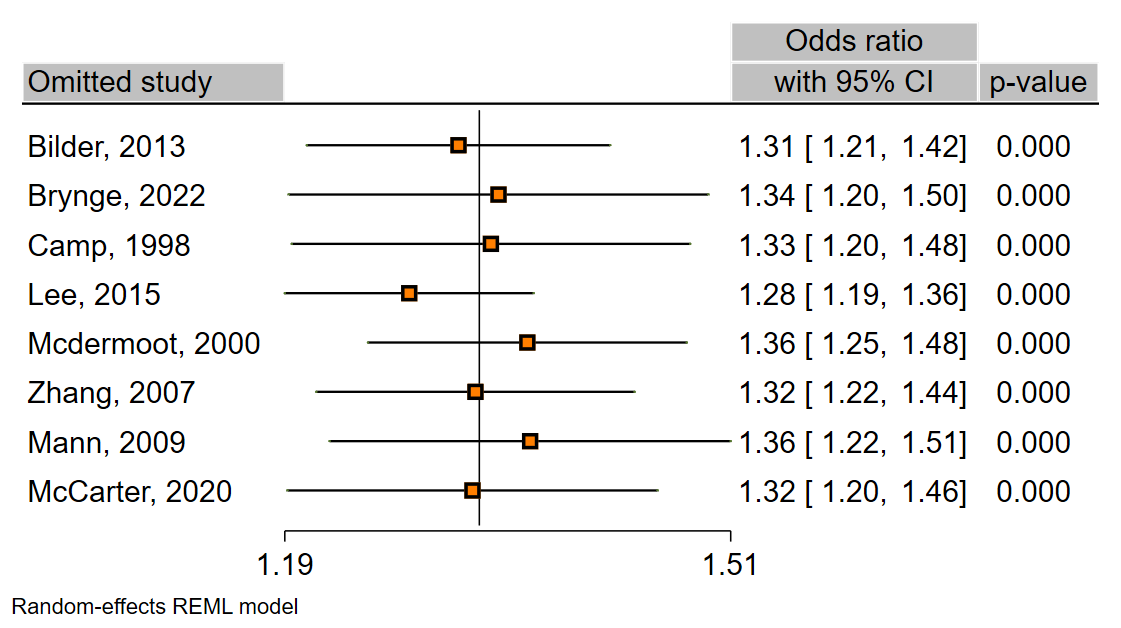

Supplement: S2 Fig — (TIF) [file pone.0292226.s003.tif]

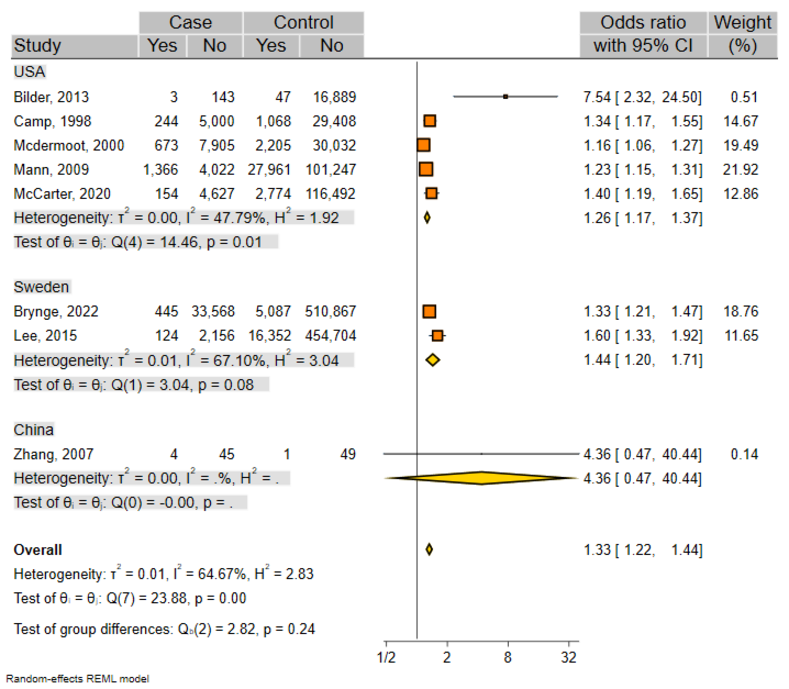

Supplement: S3 Fig — (TIF) [file pone.0292226.s004.tif]

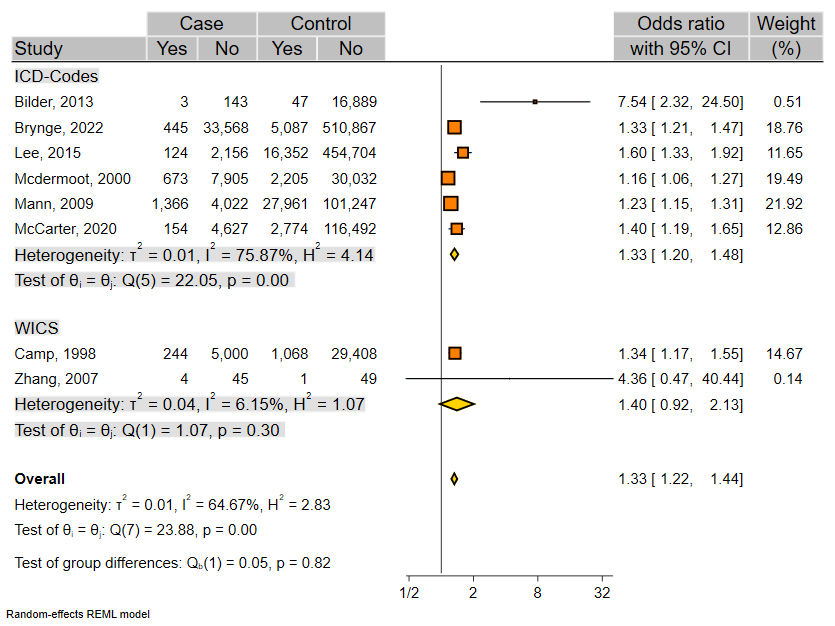

Supplement: S4 Fig — (TIF) [file pone.0292226.s005.tif]

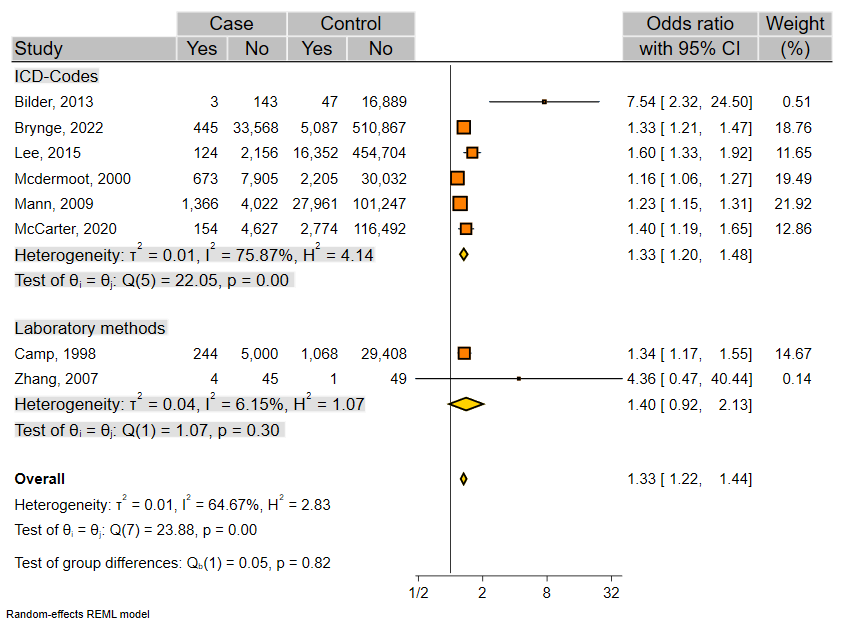

Supplement: S5 Fig — (TIF) [file pone.0292226.s006.tif]

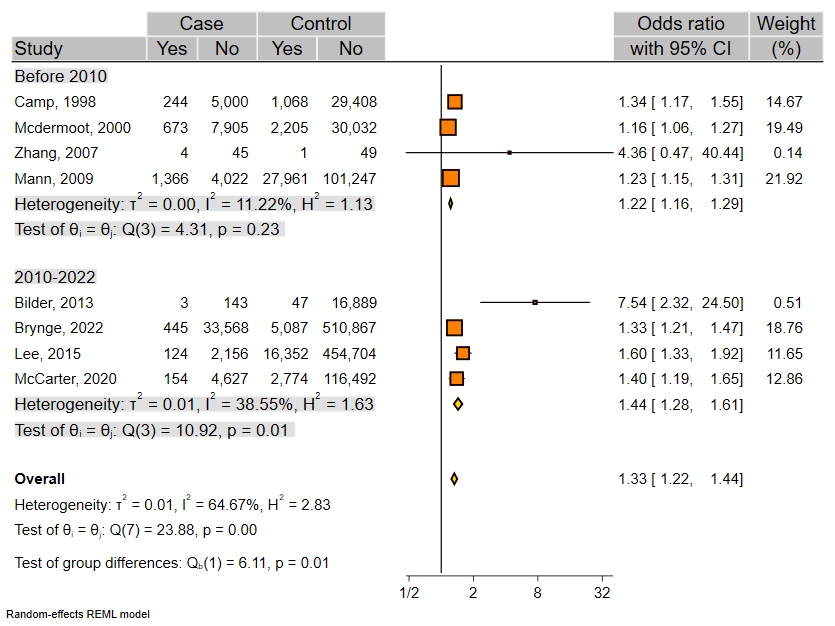

Supplement: S6 Fig — (TIF) [file pone.0292226.s007.tif]

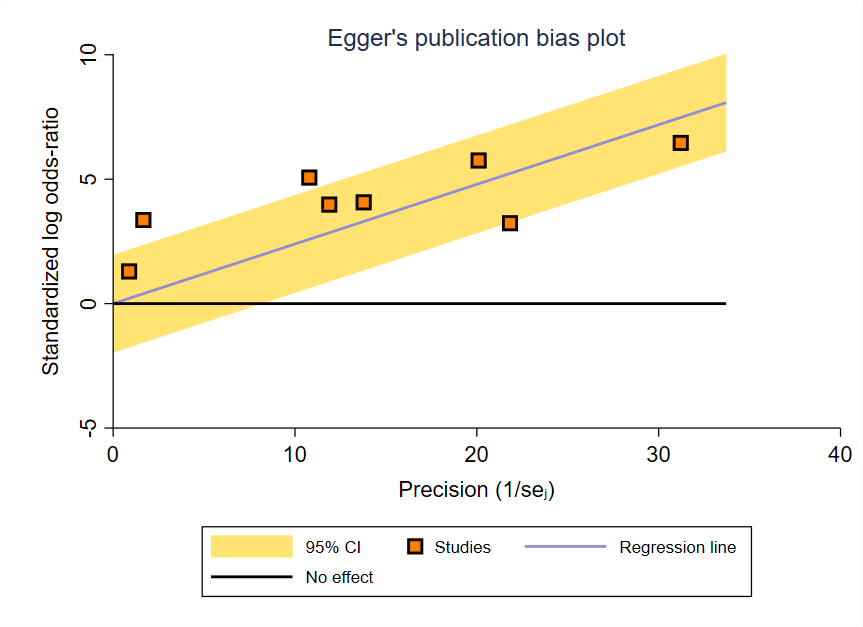

Supplement: S7 Fig — (TIF) [file pone.0292226.s008.tif]

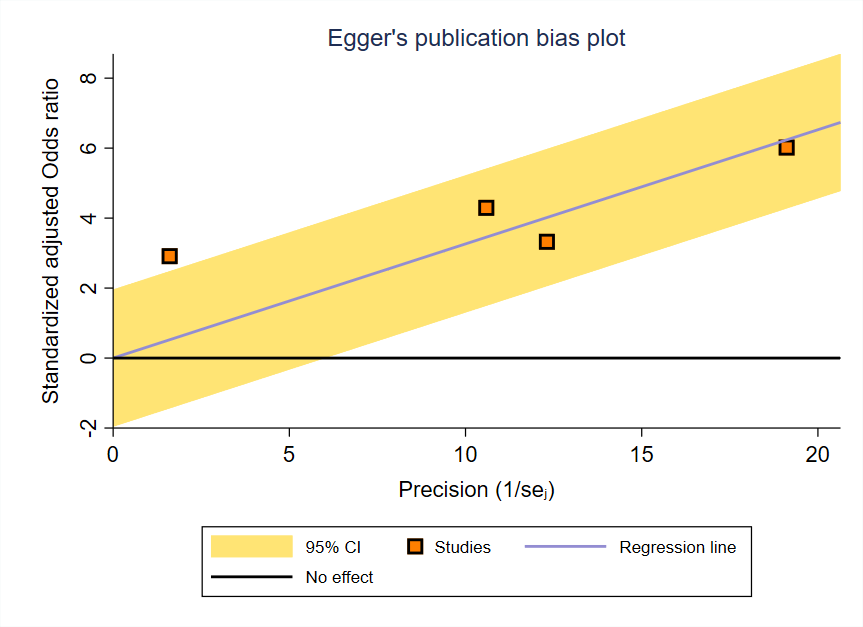

Supplement: S8 Fig — (TIF) [file pone.0292226.s009.tif]
