# Supplementary material for: The association between maternal infection and intellectual disability in children: A systematic review and meta-analysis
Source: PLoS One. 2023 Oct 5;18(10):e0292226. doi: 10.1371/journal.pone.0292226 (PMC10553326; doi:10.1371/journal.pone.0292226)
Supplement: Attachment — Submitted filename: Response to reviewers.docx [file pone.0292226.s010.docx]

**Dear Dr Yohannes Kassa**

**Academic Editor**

**Plos One**

Thank you very much for reviewing our manuscript. Comments by you and reviewers were very helpful. We revised the manuscript according to the comments raised by the you and reviewers. All the changes based on the reviewer’s comments are highlighted yellow in the revised manuscript as I explained in follow.

Sincerely yours

Additional Editor Comments :
General comments
Your methods and results sections lack clarity and need attention and revision should bed needed.

\# Abstract
In lines 30 and 31, the sentence has grammar errors and is rewritten again in an understandable way.
Line 32 and 33 also lack clarity, and it needs revision to make them clear for the reader.
How to assess publications in this meta-analysis, and you should indicate it for a reader in the abstract.
You should clearly show and convey to the readers in the methods part. It lacks clarity and is not written scientifically.
The result part also is not well written. for example, lines 38 and 39.
You can write like …… maternal infection significantly increased ID………
Lines 41 -43 also are not written clearly and need modifications.

**Response: Many thanks for your valuable suggestions. we re-written the abstract section to resolve your concerns.**

\# Results
Line 67, please state the laboratory methods they used to identify maternal infections.

**Response: Many thanks for your valuable suggestions. we added related diagnostic methods.**

In the methods section, you did not explain your outcome variable using PICO or others, which is challenging to catch infection-based and ID findings.

**Response: Many thanks for your valuable comment. We included those studies that evaluated the association between maternal infection and development of ID in children. So, we expressed the PICO specifically. The interested exposure and outcome are maternal infection and intellectual disorders, respectively. We revised the eligibility criteria as follow: “The following inclusion criteria were applied: (1) observational (cross-sectional, case-control, and cohort) studies that evaluated the association between maternal infection (interested exposure) and development of ID (interested outcome) in children (interested population)…”.**

Lines 198 -202, It is confusing the reader, and it needs to rewrite again.

**Response: Many thanks for your valuable suggestions. We re-written the paragraph to resolve your concerns.**

Please revise PRIMA, Figure 1.

**Response: Many thanks for your valuable suggestions. we edited throughout the manuscript to resolve your concerns.**

Reviewer #1: Rezaeinejad et al., has performed a meta-analysis study on a very interesting topic about Maternal infection and risk of intellectual disability in children. There are some technical and grammatical errors requiring revision from the authors. Please see my comments in details.

**Response: thanks for your valuable comments. We modified all of them.**

1- Please note that Lee et al. (2015) is not an American study, but it is performed on a Swedish population-based register study. Please correct tables and text.

**Response: thanks, addressed.**

2- The values in the following sentence “Moreover, a significant relationship was found in both infection-based- (OR, 7.10;180 95%CI, 3.50–14.38) and ID-based-(OR, 1.70; 95%CI, 0.99–2.91) studies (Table 2, Figure 2).” are different from those reported in Table 2. Please modify them.

**Response: thanks for your detailed attention, we modified them.**

3. Lines 87, 88 “The search strategy was designed and adapted it to each database by a medical library expert (Z.R).” although there is no author with these initials in the authors list.

**Response: thanks for your detailed attention, we modified them.**

4. Please use same format to write p value throughout the manuscript.

**Response: thanks, addressed.**

5. Throughout the manuscript needs to be edited for English language to modify some minor grammatical errors.

**Response: thanks, we edited throughout the manuscript.**

Reviewer #2: I read with interest this systematic review and meta-analysis about the maternal infection and risk of intellectual disability in children. The topic is interesting and the paper well written and the methodology correct.
I have some concerns:
1) The term relationship should be replaced by association, as this term is inappropriate for scientific material.

**Response: thanks, we replaced relationship by association throughout the manuscript.**

2) The Higgins index (*I^2^*), should be added to effect sizes in abstract.

**Response: thanks, we added *I*^2^ for effect size in abstract.**

3) There is controversy (typographical error) between Table 2 and results in main text and abstract especially sub-group analysis.

**Response: thanks for your detailed attention, we modified them.**

4) It is better to separate the introduction in several paragraphs not two long paragraphs. A paragraph is a group of sentences that convey an idea. Each sentence works together as part of a unit to create an overall thought or impression. A paragraph is the smallest unit or cluster of sentences in which one idea can be developed adequately. Paragraphs can stand alone or function as part of an essay, but each paragraph covers only one main idea. The most important sentence in your paragraph is the topic sentence, which clearly states the subject of the whole paragraph. The topic sentence is usually the first sentence of the paragraph because it gives an overview of the sentences to follow. The supporting sentences after the topic sentence help to develop the main idea. These sentences give specific details related to the topic sentence. A final or concluding sentence often restates or summarizes the main idea of the topic sentence.

**Response: thanks, we modified the size of paragraphs in introduction.**

5) It is better to write: Medline through PubMed not only PubMed.

**Response: thanks, addressed.**

6) As, the authors conducted a perfect sub-group analysis, it is better to omit meta-regression.

**Response: thanks, addressed.**
