# Supplementary material for: The association between maternal infection and intellectual disability in children: A systematic review and meta-analysis
Source: PLoS One. 2023 Oct 5;18(10):e0292226. doi: 10.1371/journal.pone.0292226 (PMC10553326; doi:10.1371/journal.pone.0292226)
Supplement: Attachment — Submitted filename: Response to reviewers.docx [file pone.0292226.s011.docx]

**Dear Dr Yohannes Kassa**

**Academic Editor**

**Plos One**

Thank you very much for reviewing our manuscript. Comments by you and reviewers were very helpful. We revised the manuscript according to the comments raised by the you and reviewers. All the changes based on the reviewer’s comments are highlighted yellow in the revised manuscript as I explained in follow.

Sincerely yours

Additional Editor Comments:

#Abstract
Background rewrite like this
There is arguing evidence regarding the association between maternal infections during pregnancy and the risk of intellectual disability (ID) in children. This systematic review and meta-analysis are essential to determine and address inconsistent findings between maternal infections during pregnancy and the risk of intellectual disability (ID) in children.

**R: Many thanks for your valuable corrections. We replaced your corrected sentence.**

#Methods
Line 33, from when to when and where?

**R: Thanks, we searched databases from their inception up to 15 March 2022. We modified this sentence as follow “The Medline/PubMed, Web of Science, Embase, and Scopus databases were searched from inception up to March 15, 2023, to identify potentially eligible studies.”**

Lines 35-38 you should rewrite again.

**R: We re-write these sentences again.**

#Result
Line 44, ID-based (OR, 1.70; 95%CI, 0.99–2.91; I 2 44 = 51.2) studies. It did not significantly associate with……… why you reported it here?

**R: Please accept our apologies, in previous revision we forgotten to modify these values. WE corrected them as follow:** Subgroup analysis showed a significant association for both infection-based (OR, 1.27; 95%CI, 1.15–1.40; *I*^2^ = 51.2) and ID-based (OR, 1.44; 95%CI, 1.19–1.74; *I*^2^ = 77.1) studies.

Subgroup analysis also, it is not clear to readers. What does mean infection-based and ID-based studies?

**R: we added following sentence in the method section to clarify infection-based and ID-based studies:** “The included studies were divided into two types based on the participants: (1) ID-based studies, which involved children with ID as cases and healthy children as controls and evaluated maternal infection in these participants; (2) infection-based studies, which assessed the prevalence or incidence of ID in the follow-up of children with or without exposure to maternal infection.” **Moreover, similar explanation is in main text: lines 169-175.**

#Conclusion, it needs gross modification, please pay attention to this section, you should provide a conclusion and recommendation based on your findings.

**R: we re-write this section as follow: “**Our study suggests that maternal infection during pregnancy could be associated with an increased risk of ID in children. This finding is consistent across different types of studies and diagnostic criteria. However, due to the heterogeneity and limitations of the included studies, we recommend further longitudinal studies to confirm the causal relationship and the underlying mechanisms.”

Keywords: Please use Mesh terms

**R: thanks, modified.**

#Introduction
Line 62,….. responsible for ~10.7 million years lived with disability……..it needs modifications.

**R: thanks, paraphrased it.**

Line 66-68, the sentences are out of your objectives. It focuses on maternal behaviour. but not on maternal infectious and remove it.

**R: thanks, removed.**

#Methods: state the diagnostic criteria that you included in your study?

**R: thanks, we modified related sentence as follow: “**studies that utilized universally recognized diagnostic criteria or tests to detect maternal infection or ID in children, involving International Classification of Diseases (ICD) codes or laboratory techniques (serology, molecular, or culture methods)**”.**

Why did you not use grey literatures?
Did you research using reference of referencing? if yes state it.

**R: we modified following sentence to response your above both questions: “**Furthermore, two investigators (M.J. and M.J.) searched the first 20 pages of the Google Scholar engine using the above keywords and the bibliographies of retrieved articles to find additional relevant studies and gray literature.**”**

Line 113 odds ratios (ORs), hazard ratios (HRs) or relative risks (RRs) of interested outcomes. How to be pooled it.

**R: All studies had a cohort design. All studies reported the raw counts with percentage. So, we calculated the odds ratio from the counts in contingency table. The odds ratio (OR), its standard error and 95% confidence interval are calculated according to Altman, 1991.**

**We used the following formulae to calculate the odds ratio (OR) and its confidence interval (CI).**

**OR = a*d / b*c, where:**

**a is the number of times both A and B are present,**

**b is the number of times A is present, but B is absent,**

**c is the number of times A is absent, but B is present, and**

**d is the number of times both A and B are negative.**

**To calculate the confidence interval, we use the log odds ratio, log(or) = log(a*d/b*c), and calculate its standard error:**

**se(log(or)) = √1/a + 1/b + 1/c +1/d**

**The confidence interval, ci, is calculated as:**

**ci = exp(log(or) ± Zα/2­*√1/a + 1/b + 1/c + 1/d),**

**Where Zα/2 is the critical value of the Normal distribution at α/2 (e.g. for a confidence level of 95%, α is 0.05 and the critical value is 1.96).**

**Additionally, four studies reported adjusted effect size (three studies reported odds ratio and one study reported hazard ratio). As you know there is no precise formula to convert HR to RR or OR. In short, under very specific conditions, the HR and RR will coincide when everyone has complete follow-up (no dropouts or censoring). It may be possible to compute an approximate RR by inspection of the Kaplan-Meier curves by finding the group-specific failure (or event) rates at a fixed time-point, since these are probabilities. In practical meta-analysis the researchers suppose the RR and HR identical. Odds ratios approximate risk ratios when the outcome under consideration is rare but can diverge substantially from risk ratios when the outcome is common. Logistic regression is used frequently in cohort studies and clinical trials. When the incidence of an outcome of interest is common in the study population (>10%), the adjusted odds ratio derived from the logistic regression can no longer approximate the risk ratio. The more frequent the outcome, the more the odds ratio overestimates the risk ratio when it is more than 1 or underestimates it when it is less than 1. In this meta-analysis, we considered the hazard ratio and the odds ratio to be the same.**

Line 131, if the score is how many, you included in your review?

**R: as mentioned in the result section: “Based on the methodological quality assessment scores, all studies were deemed to be of high quality.”**

Check your PRISMA.

**R: according to comment by one of the reviewers we modified PRISMA flowchart.**

In the table1, you should write types of laboratory used instead of code, for example, PCR, ELISA …… .
ID diagnostics criteria WISC……but not code

**Response: Please note that International Classification of Diseases (ICD) are most validated criteria for diagnosis of many diseases. We included related information if there were in the included studies.**

Based on your title, you should on “Infection-based studies; these studies evaluated the prevalence or incidence of ID in the follow-up of children with- and without exposure to maternal infection.” But not Intellectual disability-based studies; these studies recruited children affected by ID. because it might occur other factors like alcohol, cigarette, and others. Or modify your titles.

**Response: please note that our main was evaluate the association between maternal infection and intellectual disability. According to our consultant with epidemiologists as well as our previous experiences we can include both infection-based and ID-based of studies. However, we are agreeing with you that our title may not be suitable, therefore to resolve your concern we modified the title as follow “****The association between maternal infection and intellectual disability in children: a systematic review and meta‐analysis”**

You expected to show OR and RR of each included study in Table 2.
 **Response: Table 2 is related to sub-group analyses. Related OR for each study is presented in Figure 2.**
