# Supplementary material for: The association between maternal infection and intellectual disability in children: A systematic review and meta-analysis
Source: PLoS One. 2023 Oct 5;18(10):e0292226. doi: 10.1371/journal.pone.0292226 (PMC10553326; doi:10.1371/journal.pone.0292226)
Supplement: Attachment — Submitted filename: Response to reviewers.docx [file pone.0292226.s012.docx]

**Dear Dr Yohannes Kassa**

**Academic Editor**

**PLOS One**

Thank you very much for reviewing our manuscript. Comments by you and reviewers were very helpful. We revised the manuscript according to the comments raised by the you and reviewers. All the changes based on the reviewer’s comments are highlighted yellow in the revised manuscript as I explained in follow.

Sincerely yours

Journal Requirements:

Please review your reference list to ensure that it is complete and correct. If you have cited papers that have been retracted, please include the rationale for doing so in the manuscript text, or remove these references and replace them with relevant current references. Any changes to the reference list should be mentioned in the rebuttal letter that accompanies your revised manuscript. If you need to cite a retracted article, indicate the article’s retracted status in the References list and also include a citation and full reference for the retraction notice.

**R: Thank you very much for your inquiry. We have diligently reviewed all the articles referenced in this paper, utilizing both PubMed and the Retraction Watch website (http://retractiondatabase.org/). Regrettably, we did not uncover any retracted papers among the citations. Should you come across any retracted articles, we would greatly appreciate it if you could provide the title of the article so that we can appropriately update our reference list.**

Additional Editor Comments:

General comments

You should be expected to follow PLOS ONE guidelines.

Abstract

See punctuation.

Lines 27,32, 45, 53

**R: Many thanks for your detailed attention, we followed PLOS ONE guidelines in abstract.**

line 46: what is REM?

**R: Thanks, REM is an abbreviation for Random-effects models. We added it in Abstract**

Discussion

Line 229 -234, see again; this paragraph explains ID related to behaviours.

**R: Many thanks for your valuable comments, all your concerns were modified. We deleted this sentence and modified related sentences.**
